# Blood Pressure-Lowering Mechanisms of the DASH Dietary Pattern

**DOI:** 10.1155/2012/472396

**Published:** 2012-01-30

**Authors:** Pao-Hwa Lin, Jason D. Allen, Yi-Ju Li, Miao Yu, Lillian F. Lien, Laura P. Svetkey

**Affiliations:** ^1^Division of Nephrology, Department of Medicine, Duke University Medical Center, Durham, NC, 27710, USA; ^2^Sarah W. Stedman Nutrition and Metabolism Center, Duke University Medical Center, Durham, NC 27710, USA; ^3^Division of Cardiology, Department of Medicine, Duke University Medical Center, Durham, NC 27710, USA; ^4^Department of Biostatistics and Bioinformatics, Duke University Medical Center, Durham, NC 27710, USA; ^5^Division of Endocrinology, Metabolism and Nutrition, Department of Medicine, Duke University Medical Center, Durham, NC 27710, USA; ^6^Duke Hypertension Center, Duke University Medical Center, Durham, NC 27710, USA

## Abstract

Potential blood pressure- (BP-) lowering mechanisms of the DASH dietary pattern were measured in 20 unmedicated hypertensive adults in a controlled feeding study. At screening, participants averaged 44.3 ± 7.8 years, BMI 33.9 ± 6.6 Kg/m^2^, and BP 144.2 ± 9.38/88.5 ± 6.03 mmHg. All consumed a control diet for one week, then were randomized to control or DASH for another two weeks (week one and two). With DASH, but not controls, SBP fell by 10.65 ± 12.89 (*P* = 0.023) and 9.60 ± 11.23 (*P* = 0.039) mmHg and DBP by 5.95 ± 8.01 (*P* = 0.069) and 8.60 ± 9.13 mmHg (*P* = 0.011) at the end of week one and two, respectively. Univariate regressions showed that changes in urinary sodium/potassium ratio (*β* = 1.99) and plasma renin activity (*β* = −15.78) and percent change in plasma nitrite after hyperemia were associated with SBP changes at week one (all *P* < 0.05). Plasma nitrite following hyperemia showed a treatment effect (*P* = 0.014) and increased at week two (*P* = 0.001). Pulse wave velocity decreased over time with DASH (trend *P* = 0.019), and reached significance at week two (*P* = 0.026). This response may be mediated by an improvement in upregulation of nitric oxide bioavailability. Early natriuresis and reductions in oxidative stress cannot be ruled out. Future studies are needed to verify these findings, assess the possibility of earlier effects, and examine other potential mediators.

## 1. Introduction

Randomized, controlled feeding trials have established the blood pressure- (BP-) lowering effects of the Dietary Approaches to Stop Hypertension (DASH) dietary pattern [1, 2], which emphasizes fruits, vegetables, and low-fat dairy and is low in meats, sugar-sweetened beverages, and saturated and total fat. These original DASH studies were designed to establish efficacy, not to determine the mechanism of action. Postulated mechanisms for the BP-lowering effect of the DASH dietary pattern include effects on the natriuresis, renin-angiotensin-aldosterone system (RAAS), reduced adrenergic tone, and increased vascular relaxation [3–6]. To date, these mechanisms have not been directly evaluated. An understanding of the mechanism(s) by which the DASH dietary pattern lowers BP can potentially enhance the efficacy of this dietary intervention by elucidating the conditions under which it will be most effective and help to identify target populations who may receive optimal benefits. It may also help to define interactions or synergistic effects with other concurrent BP interventions such as pharmacotherapy.

Therefore, the overall goal of this pilot project was to determine the mechanism(s) by which the DASH dietary pattern lowers BP in unmedicated hypertensive individuals.

## 2. Patients and Methods

### 2.1. Study Design

**Figure 1:**
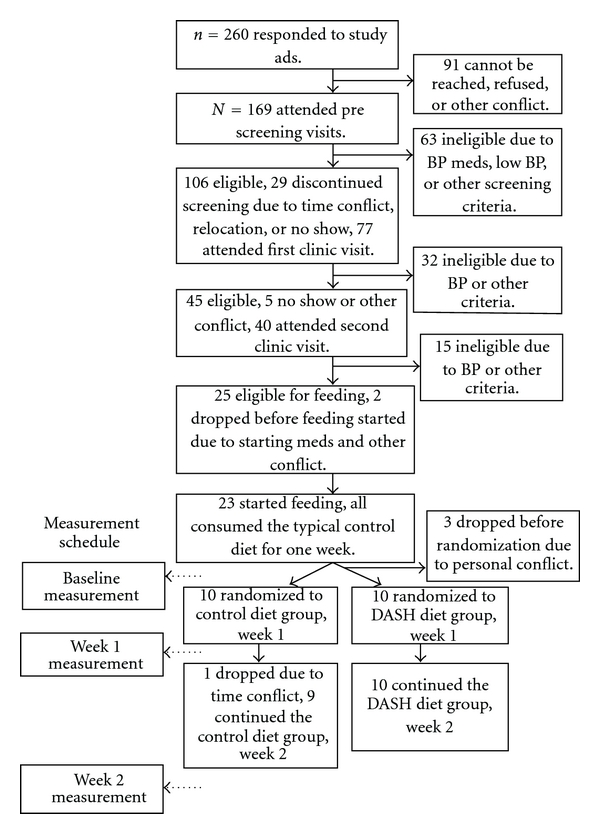
Design and flow of study.

This study was a randomized controlled feeding trial testing the effects of two dietary patterns on sodium excretion, plasma renin activity, aldosterone, catecholamines, and markers of vascular endothelial function. The design and flow of the study is illustrated in Figure 1. The study was approved by the Duke University Medical Center Institutional Review Board, and all participants provided written informed consent prior to participation. After screening, participants started the controlled feeding protocol. For the first week (run-in), all participants consumed a typical American (control) diet (Table 1). Participants were then randomly assigned to either continue with the control diet or switch to the DASH dietary pattern. Since the BP-lowering effect of the DASH dietary pattern is maximal after two weeks of controlled feeding [1], participants in the current trial ate the assigned dietary pattern for two weeks after randomization. Outcomes were measured at the end of run-in and at the end of week one and week two of the randomized feeding.

### 2.2. Study Participants

Participants were included in the study if they were age 22 yrs or older, stage 1 hypertensive (average SBP 140–159 and DBP 90–99 mmHg over two screening visits), not taking BP medication for at least 3 months prior to screening, had a BMI within 18.5–40 kg/m^2^, not pregnant or nursing, and willing to be randomized to either of the treatment groups. Exclusion criteria included secondary hypertension, diabetes requiring insulin or hypoglycemic agents, special dietary requirements incompatible with study diets, comorbid medical condition such as active cancer, heart or lung disease, kidney disease (estimated GFR < 60 mL/min), or other major illness, consuming more than 14 alcoholic drinks per week, or major psychiatric disorder. All potential participants completed a phone pre-screening interview first to establish major eligibility criteria, and then attended two face-to-face screening visits to further establish eligibility and obtain pre-run-in measures.

### 2.3. Intervention

#### 2.3.1. Content of the Dietary Patterns

The nutrient profiles of the two diets are presented in Table 1 and were consistent with previous DASH trials. Specifically, the DASH dietary pattern emphasized fruits, vegetables, and low-fat dairy foods, included whole grains, poultry, fish, and nuts and was reduced in fats, red meat, sweets, and sugar-containing beverages [7]. Relative to the typical American control diet, DASH was reduced in total and saturated fat and cholesterol and increased in potassium, calcium, magnesium, dietary fiber, and protein. The composition of the control diet was based on the profile reported in the NHANES III survey [8] and contained relatively high total and saturated fat and cholesterol and lower fruits, vegetables, and low fat dairy products. In order to isolate the effects of the DASH dietary pattern, sodium intake for both diets was the same (3400 mg/2100 kcal/day), comparable to typical US consumption as estimated by NHANES III.

This study employed the same controlled feeding protocol and followed the same quality assurance procedures used in the DASH and the DASH-sodium multicenter trials [1, 2]. All foods and beverages for each participant were purchased and prepared in the metabolic kitchen at the Sarah W. Stedman Nutrition and Metabolism Center. As in the DASH studies, participants consumed the main meal of the day at the Stedman Center from Monday to Friday under the supervision of the study staff and took home the rest of the foods as “to go” packages. Adherence was assessed by direct observation by the supervising staff during on-site meals and by a questionnaire completed daily on which participants indicated if they failed to eat any of the “take home” study foods or ate any foods that were not provided by the study.

At the beginning of the study, energy (calorie) requirement for each participant was estimated by using the WHO formula adjusted with a physical activity factor estimated from a seven-day physical activity questionnaire [9]. During feeding, body weight was monitored daily, and caloric intake was adjusted quickly to maintain constant weight during the entire study period.

Randomization occurred electronically in the middle of the run-in period to allow time for kitchen staff to prepare foods. Participants were not notified of their randomization assignment but were likely to infer it when they received the first meal of the randomized feeding. The inability to fully blind study participants to treatment assignment is a known and unavoidable limitation of most feeding studies.

### 2.4. Measurements

All study measurements were performed by trained, certified study personnel during face-to-face clinic visits. The study personnels who obtained these measurements were blinded to intervention assignments. BP was measured in a standardized manner, using an automated device [1]. At the beginning of each visit, participants rested quietly for five minutes in the seated position with a back rest. An appropriately sized cuff was applied to the right arm after measurement of arm circumference. The automatic sphygmomanometer device was activated, and a BP measurement was recorded. Second and third readings were obtained, each after a 30-second rest period. The last two BP measurements from each visit were averaged to determine BP at the end of run-in (baseline) and subsequent follow-up visits. Body weight was measured in light indoor clothes without shoes to the nearest 0.1 kg using a high-quality digital scale. 

Dietary intake prior to the study was assessed by collecting a four-day food record during screening, a commonly used reference method for diet assessment [10, 11], and the records were analyzed with the Nutrition Data System (NDSR). Twenty-four-hour urine collections were aliquoted and sent to LabCorp for assessment of urinary sodium (Na^+^), potassium (K^+^), calcium (Ca^++^), magnesium (Mg^++^), phosphorous (P), creatinine, and urea nitrogen excretion. In steady state, urinary K^+^, P, and urea nitrogen serve as markers of dietary intake of fruits and vegetables, dairy, and protein, respectively. Potential mediators of DASH BP response were measured after an overnight fast (except for 24-hour urine collections) and included the following.

(i) Natriuresis was assessed by 24-hour urinary excretion of Na^+^.

(ii) Plasma renin activity (PRA) and aldosterone were assayed in a high-quality commercial clinical lab (LabCorp) from fasting blood samples collected after participants were in sitting position for at least 15 minutes.

(iii) Urinary catecholamines (epinephrine and norepinephrine) were analyzed from the 24-hour urinary specimen by HPLC with electrochemical detection in a research laboratory at Duke [12].

(iv) Plasma nitric oxide (NO) metabolite analysis was performed on blood taken from the median-cubital vein in the test arm at rest and 60 sec following an upper arm occlusion for 5 minutes. Briefly, the blood samples were drawn into an untreated syringe and mixed with 1 : 1000 heparin at a ratio of 1 mL blood to 5 uL heparin. The plasma was separated out and frozen in liquid nitrogen immediately following collection and then stored at −80°C until assayed for various NO metabolites by reductive chemiluminescence [13].

(v) Vascular endothelial function was measured by brachial artery flow-mediated dilation (BAFMD). All vascular imaging was performed between 8 am and 11 am, on the left arm, with the participant in a supine position with the forearm extended and slightly supinated. Brachial artery assessments were obtained using high-resolution ultrasound and a 7.5 MHz linear array transducer (Acuson, Sequoia 512), at baseline (following 10 min of supine rest), during five minutes of forearm occlusion and continuously on r-wave trigger for 2 minutes following cuff release (hyperemia) as previously described [14, 15]. Arterial diameter and blood flow were measured from the digital recordings. Diameters were determined from the anterior to posterior interface between the intimal layers. The percent change in brachial artery diameter was calculated by the following formula:

(1) $$\begin{array}{c} \left(\frac{\text{peak}\;\text{posthyperemia}\;\text{diastolic}\;\text{diameter}-\text{baseline}\;\text{diastolic}\;\text{diameter}}{\text{baseline}\;\text{diastolic}\;\text{diameter}}\right)*100.\; \end{array}$$

Reproducibility of the BAFMD technique has previously yielded average mean differences in brachial artery diameter change for days, testers, and readers of 1.91%, 1.40%, and 0.21 mm, respectively, with intraclass correlation coefficients of 0.92, 0.94, and 0.90, respectively [15].

(vi) Vascular Stiffness was measured by utilizing radial artery pulse wave analysis (RAPWA) and carotid-posterior tibial pulse wave velocity (PWV). All artery pressure waveforms were measured using a Millar tonometer and processed using dedicated software (SphygmoCor version 8.0, AtCor Medical). The system was used to measure an averaged radial artery waveform, derive a corresponding central aortic pressure waveform [16], then calculate pressure augmentation, augmentation index (AIx), and pulse pressure amplification [17]. For PWV assessment, body surface distances from the suprasternal notch to the carotid and tibial sites were measured. Sequential measurements of arterial pressure waves at these two sites were then made using the foot-of-the-wave method [18]. The transit times of each wave were calculated from the onset of the electrocardiogram QRS complex.

### 2.5. Statistical Analysis

All analyses were performed by using the SAS software (v.9.2, SAS Institute, Cary, NC, USA). Initially, primary outcome data (SBP and DBP) were tested for normality using quantile-quantile plot. Background characteristics were compared between the two treatment groups by using *t*-test if continuous variables or Fisher's exact test if categorical data. Three analytical strategies were subsequently used: First, changes over time between the two treatment groups were examined by repeated measure MANOVA tests and comparison between treatments or visits were further conducted when there is a significant time (visit) or treatment effect. Second, changes over time were examined by trend analysis within each treatment group. Lastly, simple univariate regression was used to explore the relationship between changes in each variable with changes in BP. Variables that showed a significant association in the simple regression were then included in a multivariate model. Data collection was complete for all but one of the twenty participants, who discontinued after the week one assessment. BP and other outcome data from this participant were carried forward from week one to the end of week two. The significance level was set at *P* < 0.05. All data were presented as mean ± SD.

## 3. Results

A total of 20 adult participants with unmedicated stage 1 hypertension completed the study. They averaged 44.3 ± 7.8 yrs of age, had a BMI of 33.9 ± 6.6 Kg/m^2^(consistent with class I obesity), BP of 144.2 ± 9.38/88.5 ± 6.03 mmHg (Table 2). Among the participants, 13 were females, five were self-identified Caucasians, 14 were African Americans, and one was Hispanic. During screening, these participants reported consuming an average of 1 alcoholic beverage per week. Despite small sample size, Table 2 demonstrates that randomized groups were fairly comparable particularly regarding baseline BP. However, participants in the control group were younger and heavier in weight (both *P* < 0.05).

**Figure 2:**
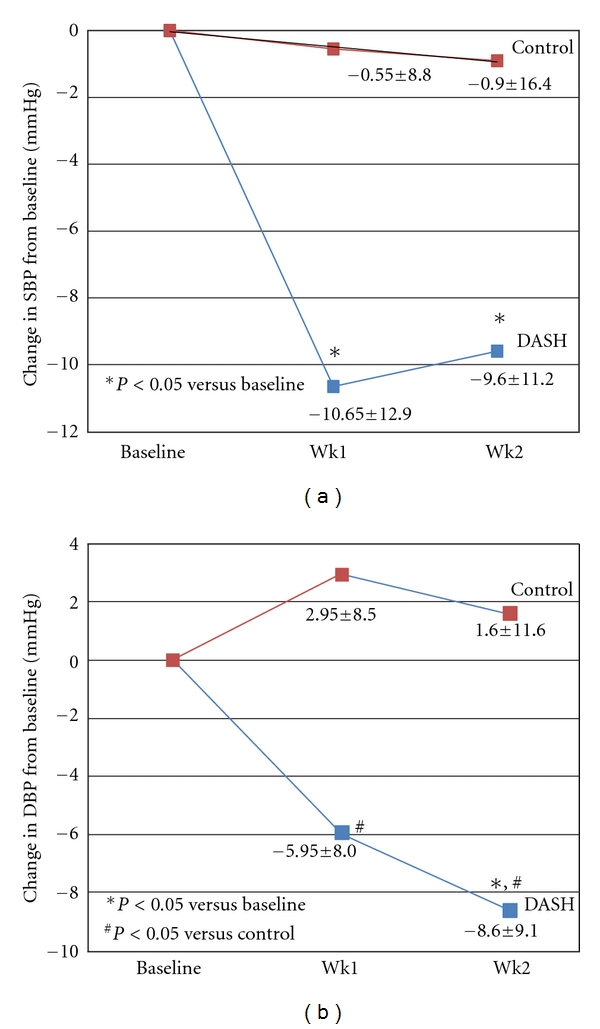
Blood pressure changes from baseline by treatment.

The MANOVA test indicates that there were significant treatment (dietary pattern) effects on both SBP and DBP (*F* = 5.46, *P* = 0.023 and *F* = 9.73, *P* = 0.003, resp.). As shown in Figure 2, DASH, comparing to baseline, significantly reduced SBP at week one (mean change from baseline −10.65 ± 12.89 mmHg, CI : −19.81, −2.49, *P* = 0.023), with sustained effect at week two (−9.60 ± 11.23 mmHg, CI : −16.70, −2.50, *P* = 0.039). Similarly, DASH lowered DBP to a near-significant degree at week one (−5.95 ± 8.01 mmHg, CI : −11.02, −0.88, *P* = 0.069) as compared to the baseline, with statistically significant reduction at week two (−8.60 ± 9.13 mmHg, CI : −14.38, −2.82, *P* = 0.011). Both SBP and DBP remained constant with exposure to the control dietary pattern. The difference between DASH and control approached statistical significance for SBP at week one only (*P* = 0.056) and were statistically significant for DBP at both week one and week two (*P* = 0.027 and *P* = 0.043, resp.).

As expected based on the nutrient composition of diets, urinary K^+^, Mg^++^, and P increased in the DASH group and remained relatively unchanged in the controls (Table 3). Urinary K^+^ increased significantly over time with DASH and the increases were significantly different from that of the controls. The increases in urinary Mg^++^ and P in the DASH group were significantly greater than that of the controls at week one. These findings confirmed adherence to the assigned dietary patterns. However, despite increased dietary Ca, urinary Ca^++^ did not change with the DASH dietary pattern, similar to findings of previous DASH studies [1].

### 3.1. Potential Mechanisms or Mediators

(i) Natriuresis. Urinary Na^+^ remained relatively constant in both groups (although, however, urinary Na^+^/K^+^ ratio decreased significantly in the DASH group at both week one and two as compared to the baseline (Table 4)).

(ii) RAAS. Overall, changes in PRA showed a significant treatment effect (MANOVA *P* = 0.004). Table 4 indicates that PRA showed a tendency to increase in the DASH group while decreasing in the control group (both NS). The change in PRA was significantly different between the two treatment groups at week one (*P* = 0.022), but the difference was attenuated at week two (*P* = 0.071). Similarly, plasma aldosterone tended to increase in the DASH group, but there were no significant differences between the treatment groups.

(iii) Adrenergic tone. Neither urinary norepinephrine nor epinephrine showed any changes during the study. 

(iv) Vascular measurements. Among all the vascular measurements, plasma nitrite (NO_2_^−^) level following hyperemia showed a significant treatment effect (*P* = 0.014) in MANOVA (Table 4). Plasma nitrite (NO_2_^−^) after hyperemia started to show an increase at week one as compared to the baseline, and the increase was significant at week two (*P* = 0.001). This increase was greater than that of the controls at week two (*P* = 0.053). This pattern was also observed in the change of plasma nitrite after hyperemia and that the change in DASH increased significantly at week two (*P* = 0.053), whereas there was a significant decrease within the controls over time (*P* = 0.026). Similar pattern was observed in the percent change in plasma nitrite after hyperemia. Neither BAFMD nor AIx changed during the course of the study. PWV decreased over time in the DASH group (trend *P* = 0.019), and the reduction at week two was significant compared to baseline (*P* = 0.026). 

In simple linear univariate regressions (Table 5), changes in urinary Na^+^ (*β* = 0.12), K^+^ (*β* = −0.19), Na^+^/K^+^ ratio (*β* = 1.99), and PRA (*β* = −15.80) showed a significant association with changes in SBP at week one (all *P* < 0.05). Percent change in plasma nitrite after hyperemia also showed a significant negative association with change in SBP (*β* = −0.301, *P* = 0.035). When the urinary Na^+^/K^+^ ratio, PRA, and percent change in plasma nitrite after hyperemia were included in a multivariate regression model, all three variables remained significantly associated with SBP change (*P* < 0.05). Similar pattern is observed with DBP change (data not shown). 

## 4. Discussion 

Our data suggest that DASH may lower BP by increasing NO bioavailability, as measured by plasma nitrite following a stressor, and possibly has subsequent effects on vascular basal tone (reduced PWV). There was no evidence that BP reduction was due to effects of DASH on natriuresis, suppression of the RAAS (as reflected in changes in PRA and aldosterone), or on adrenergic tone (as reflected in changes in epinephrine and norepinephrine). Due to small sample size and other limitations discussed below, these data should be considered preliminary evidence necessitating further study. 

Although our previous research showed that DASH may shift the pressure-natriuresis curve [3] and that potassium supplementation leads to natriuresis [19], we saw no clear evidence of natriuresis. However, the rise in both PRA and aldosterone suggests a natriuretic effect of DASH. This is consistent with previous studies that showed an increase in PRA in response to a high intake of K^+^, a rich component in DASH [20, 21]. Further, our data suggest that BP-lowering occurred prior to the week one assessment. Therefore, it is possible that natriuresis occurred earlier than that assessment and had reequilibrated by the end of week one. In fact, potassium supplementation causes natriuresis within four days [19]. Therefore, we cannot rule out the possibility that natriuresis contributes to the BP-lowering effect of DASH, and future studies are needed in which urinary Na excretion is measured immediately after initiating the DASH dietary pattern. 

Our data do not provide evidence that DASH lowers BP by direct effects on the RAAS. Renin activity, the rate-limiting step in generation of the vasoconstrictor angiotensin II, was actually increased relative to changes in the control group, possibly due to compensation for lower BP. We did not measure other RAAS mediators that might reveal a role of the RAAS; indeed, we know that a polymorphism of the angiotensinogen gene is associated with the BP response to DASH [22]. Therefore, a role for the RAAS in mediating BP lowering with DASH cannot be ruled out, at least in some genetically predisposed individuals. 

Our data also provide no evidence that DASH inhibits the adrenergic nervous system. Neither urinary norepinephrine nor epinephrine changed with DASH, but these are fairly crude measures of adrenergic tone. Changes in adrenergic tone can be episodic, and multiple urine collections may be necessary to reflect the change. Indeed, there is evidence of genetic modulation of the DASH effect by a polymorphism of the *β*2-adrenergic receptor gene [22]. Thus an effect of DASH on adrenergic nervous system regulation of BP cannot be ruled out. 

The increases in stimulated plasma nitrite in DASH relative to controls suggests that the DASH dietary pattern may improve the vascular endothelium's ability to up-regulate NO, in response to a localized stressor. The DASH dietary pattern contains nuts which are a good source of L-arginine, a precursor of nitric oxide (NO) [23, 24]. Oxidative stress and endothelial dysfunction have been implicated in hypertension [25]. Previous studies have also shown that foods/diets rich in antioxidants, L-arginine, and marine n-3 fatty acids, such as DASH, may also improve vascular reactivity [26–28]. In a behavioral intervention study, following the DASH dietary pattern enhanced antioxidant capacity and prevented worsening of endothelial function during acute hyperlipidemia induced by intralipid infusion among obese hypertensives [29]. The authors attributed the vascular protection effect to the rich antioxidants in the DASH dietary pattern. In our study we did not observe an increase in BAFMD following the DASH diet. This may be related to the limited sample size in each group and relatively short assessment period. Another trial also did not observe an impact of consuming the DASH diet for 30 days on vascular function including measurement of FMD [30]. However, dietary adherence of these participants to the DASH pattern was not reported. A recent study, nevertheless, showed that consuming the DASH diet for 3 weeks improved endothelial function as indicated by small artery elasticity [31]. The changes in plasma nitrite (the primary metabolite of vascular NO production) suggest an increase in NO bioavailability and may change prior to the physiological response. It is possible that the BP-lowering effect of DASH could be mediated through its effects of antioxidants on NO consumption. In a high antioxidant environment, it is possible that more NO produced would be oxidized to nitrite rather than nitrate and peroxynitrite. In addition, effects of DASH on NO bioavailability and metabolism may have implications for cardiovascular health aside from just BP control. Future studies should examine the direct impact of the DASH dietary pattern on vascular health. 

The possibility that the BP-lowering effect of DASH may be mediated through its impact on the vascular endothelium is further suggested by the observed reduction in PWV. Previous research has shown that PWV is an independent predictor of increasing SBP over time and of incident hypertension among people with normotension or untreated hypertension, respectively [32]. Our study suggests that arterial stiffening, as indicated by PWV, may be an underlying cause of the rise in BP. In addition, PWV was found to be a strong and independent predictor of CVD mortality with high performance values among hypertensive and diabetic patients [33, 34]. For every 1 m/s increase in PWV, the hazard ratio for all-cause and CVD mortality increases by 8% [34]. Thus, even if the impact of the DASH dietary pattern on PWV was not related to the mechanism for BP reduction, its potential benefit on CVD mortality via this marker deserves further examination and confirmation. 

The urinary Ca^2+^ data may suggest another possible BP-lowering mechanism. As noted above, urinary Ca^2+^ remained unchanged in both treatment groups despite a much higher intake in the DASH group than in the controls. Our previous study showed that the combined effects of higher potassium and low acid load of the DASH diet may contribute to Ca retention and subsequent reduced bone turnover [35]. Thus conservation of Ca likely occurred within the DASH group, which may have contributed to the BP reduction through vascular effects of Ca. This possibility also deserves further investigation. 

This study is limited primarily by the small sample size. A controlled feeding protocol, which was used in, and is one of the strengths of, this study allows for near-absolute control over nutrient intake in humans, eliminating extensive confounding that occurs in studies in which participants select and prepare their own meals. However, feeding studies are limited by the resources required and participant burden. Nonetheless, large feeding studies with adequate power are feasible [1, 2, 36]. Our data suggest that such a study would be valuable in definitively determining effects of DASH on NO bioavailability, oxidative stress, antioxidant capacity, as well as effects on other BP mediators in the RAAS on the adrenergic nervous system, and at earlier time points when BP is going down. 

This study sheds light on the possible mechanism(s) through which the DASH dietary pattern lowers BP. Confirmation in larger populations, evaluation of other potential mediators, and a deeper understanding of molecular mechanisms and pathways are needed. Understanding the BP-lowering mechanisms of DASH can lead to more refined nutritional recommendations, the identification of new drug targets, greater insight into normal and abnormal BP regulation, and more targeted treatments for individual patients.

## Figures and Tables

**Table 1:** Nutrient composition of the two dietary assignments.^1^

Nutrients	DASH	Control
Energy (kcal)	2100	2100
Total fat (% kcal)	27	34
Saturated fatty acids (% kcal)	4	13
Monounsaturated fatty acids (% kcal)	15	13
Polyunsaturated fatty acids (% kcal)	8	8
Protein (% kcal)	18	15
Carbohydrate (% kcal)	56	49
Cholesterol (mg)	150	300
Fiber (g)	32	11
Potassium (mg)	4700	1700
Magnesium (mg)	500	160
Calcium (mg)	1250	450
Sodium (mg)	3400	3400

^1^All data based on daily energy intake of 2100 kcal. Percentages for macronutrients (fats, protein, and carbohydrate) remained the same for all caloric levels. Other target nutrients including fiber, potassium, magnesium, calcium, and sodium were proportional to caloric intake.

**Table 2:** Baseline characteristics of study participants.

Characteristics^1^	All	Control	DASH
*N*	20	10	10
Age, yr	44.3 ± 7.8	42.4 ± 6.3	46.1 ± 9.0*
Female, *n *	13	6	7
Race, *n *			
Caucasian	5	2	3
African American	14	8	6
Other	1	0	1
BMI, kg/m^2^	33.9 ± 6.6	36.9 ± 6.1	31.0 ± 5.9*
Alcohol, serv./wk	1.0 ± 2.3	1.1 ± 2.7	0.9 ± 1.9
SBP, mmHg (screening)	144.2 ± 9.38	145.6 ± 11.5	142.8 ± 7.03
DBP, mmHg (screening)	88.5 ± 6.03	88.2 ± 5.68	88.8 ± 6.67

^1^Mean ± SD. *P < 0.05 comparing to the control group using *t*-test for continuous variables and the Fisher exact test for categorical data.

**Table 3:** Adherence measures by treatment and time.

Measurement Mean ± SD	Control	DASH	*P* for DASH versus control
*Urinary excretion*			
Potassium, mmol/24 hr			
Baseline	46.6 ± 13.3	42.2 ± 14.7	
Change at week 1	−10.2 ± 11.9	39.8 ± 25.8*	<0.0001
Change at week 2	−7.9 ± 16.4	25.3 ± 28.6^†^	0.005
*P* for trend	0.367	0.075	

Magnesium, mmol/24 hr		
Baseline	3.56 ± 1.54	3.51 ± 1.20
Change at week 1	0.22 ± 1.01	1.32 ± 0.86^‡^	0.017
Change at week 2	−0.01 ± 1.07	0.31 ± 1.27	0.553
*P* for trend	0.819	0.454	

Phosphorus, mmol/24 hr		
Baseline	251.8 ± 78.82	222.6 ± 61.22
Change at week 1	7.53 ± 24.94	82.68 ± 112.4	0.054
Change at week 2	39.44 ± 120.6	−35.76 ± 114.7	0.170
*P* for trend	0.218	0.446	

Urea nitrogen, mol/24 hr		
Baseline	3.73 ± 1.26	3.44 ± 0.95
Change at week 1	−0.57 ± 1.01	0.29 ± 1.04	0.077
Change at week 2	−0.63 ± 1.32	−0.26 ± 1.26	0.524
*P* for trend	0.629	0.780	

Calcium, mmol/24 hr		
Baseline	36.06 ± 25.83	39.92 ± 23.71
Change at week 1	3.75 ± 16.79	−5.50 ± 12.75	0.182
Change at week 2	0.55 ± 14.76	−7.51 ± 13.41	0.218
*P* for trend	0.909	0.298	

Sodium, mmol/24 hr		
Baseline	122.6 ± 40.82	131.4 ± 42.83
Change at week 1	19.3 ± 33.39	−4.2 ± 57.49	0.278
Change at week 2	17.85 ± 65.93	−1.5 ± 64.40	0.620
*P* for trend	0.493	0.919	

*P = 0.0004, ^†^ *P* = 0.017, ^‡^ *P* = 0.002 compared to baseline within treatment group using the MANOVA analysis.

**Table 4:** Measures of potential mechanisms by treatment and time.

Measurement Mean ± SE	Control	DASH	*P* for DASH versus control
Urinary sodium/potassium ratio			
Baseline	2.76 ± 1.13	3.20 ± 0.69	
Change at week 1	1.23 ± 2.28	−3.54 ± 3.52*	0.002
Change at week 2	0.61 ± 1.20	− 3.06 ± 2.91^†^	0.004
*P* for trend	0.112	0.018	

Plasma renin activity, *μ*g/L·hr			
Baseline	0.74 ± 1.03	0.44 ± 0.28	
Change at week 1	−0.21 ± 0.30	0.38 ± 0.63	0.022
Change at week 2	−0.11 ± 0.24	0.22 ± 0.46	0.071
*P* for trend	0.653	0.441	

Plasma aldosterone, nmol/L			
Baseline	0.35 ± 0.24	0.32 ± 0.10	
Change at week 1	−0.04 ± 0.19	0.04 ± 0.16	0.361
Change at week 2	0.02 ± 0.22	0.04 ± 0.17	0.846
*P* for trend	0.232	0.727	

Urinary norepinephrine/Cr, *μ*mol/mol			
Baseline	11.38 ± 5.27	9.39 ± 3.76	
Change at week 1	−0.28 ± 3.01	0.97 ± 4.02	0.440
Change at week 2	2.87 ± 14.50	1.95 ± 4.64	0.851
*P* for trend	0.829	0.525	

Urinary epinephrine/Cr, *μ*mol/mol			
Baseline	1.44 ± 0.73	1.18 ± 0.47	
Change at week 1	0.07 ± 0.78	0.21 ± 0.60	0.668
Change at week 2	−0.14 ± 0.74	0.60 ± 1.75	0.231
*P* for trend	0.713	0.243	

NO_2_^−^ before hyperemia, *μ*mole			
Baseline	286.3 ± 165.0	279.6 ± 188.5	
Change at week 1	54.36 ± 91.04	91.6 ± 166.1	0.564
Change at week 2	67.9 ± 141.9	65.5 ± 195.3	0.976
*P* for trend	0.696	0.163	

NO_2_^−^ after hyperemia, *μ*mole			
Baseline	317.4 ± 181.1	241.7 ± 166.6	
Change at week 1	−1.90 ± 69.92	43.59 ± 53.63	0.141
Change at week 2	5.92 ± 103.9	93.94 ± 71.78^‡^	0.053
*P* for trend	0.675	0.036	

NO_2_^−^ change (absolute), *μ*mole			
Baseline	31.04 ± 60.05	−37.85 ± 64.89	
Change at week 1	−56.26 ± 59.15	−47.98 ± 144.7	0.141
Change at week 2	−62.02 ± 72.5	28.48 ± 164.7	0.053
*P* for trend	0.026	0.423	

NO_2_^−^ change, %			
Baseline	12.16 ± 25.30	−14.84 ± 15.25	
Change at week 1	−19.63 ± 13.48	−0.80 ± 23.41	0.053
Change at week 2	−14.11 ± 37.65	18.12 ± 38.89	0.093
*P* for trend	0.004	0.173	

BAFMD peak % change,%			
Baseline	5.20 ± 3.28	6.07 ± 3.60	
Change at week 1	1.64 ± 1.36	−0.32 ± 3.99	0.159
Change at week 2	1.47 ± 3.56	−0.41 ± 4.94	0.341
*P* for trend	0.310	0.741	

Augmentation index, %			
Baseline	22.43 ± 11.21	19.0 ± 9.68	
Change at week 1	4.17 ± 6.68	1.0 ± 5.48	0.348
Change at week 2	3.0 ± 5.51	2.63 ± 6.26	0.905
*P* for trend	0.717	0.270	

PWV, m/s			
Baseline	9.87 ± 2.72	10.23 ± 1.65	
Change at week 1	−0.24 ± 2.22	−0.70 ± 1.99	0.681
Change at week 2	−0.47 ± 1.87	−1.54 ± 2.39^‡‡^	0.358
*P* for trend	0.437	0.019	

*P = 0.006, ^†^ *P* = 0.015, ^‡^ *P* = 0.001,^ ‡ ‡^ *P* = 0.026 compared to baseline within treatment group using the MANOVA analysis.

**Table 5:** Simple regression of SBP changes on changes in individual biological and vascular variables at week one.

Variable	Week one	
	Slope	*P* value
Urinary sodium	0.123	0.030
Urinary potassium	−0.194	0.018
Urinary sodium/potassium ratio	1.987	0.003*
Urinary norepinephrine/creatinine	−685	0.197
Urinary epinephrine/creatinine	−2256	0.376
Plasma renin activity	−15.80	0.0003*
Plasma aldosterone	−0.473	0.312
Plasma nitrite after hyperemia, %	−0.301	0.035*
Brachial artery flow-mediated dilation	0.021	0.982
Augmentation index, %	0.736	0.174
Pulse wave velocity, m/s	−0.184	0.905

*Since urinary sodium and urinary potassium are closely related with urinary sodium/potassium ratio, only urinary sodium/potassium ratio, plasma renin activity, and % change in plasma nitrite after hyperemia were further included in a multivariate regression model. All three variables remained significantly associated with SBP change at week one (*P* < 0.05 for all).
